# Cofactor-mediated conformational control in the bifunctional kinase/RNase Ire1

**DOI:** 10.1186/1741-7007-9-48

**Published:** 2011-07-06

**Authors:** Alexei V Korennykh, Pascal F Egea, Andrei A Korostelev, Janet Finer-Moore, Robert M Stroud, Chao Zhang, Kevan M Shokat, Peter Walter

**Affiliations:** 1 Howard Hughes Medical Institute, University of California, San Francisco, 600 16th Street, Room S272, Box 0724, San Francisco, CA 94158, USA; 2 Department of Biochemistry and Biophysics, University of California, San Francisco, Genentech Hall, 600 16th Street, San Francisco, CA 94158, USA; 3 Department of Biochemistry and Molecular Pharmacology and RNA Therapeutics Institute, University of Massachusetts Medical School, 364 Plantation Street, Worcester, MA 01605, USA; 4 Department of Cellular and Molecular Pharmacology, University of California, San Francisco, Genentech Hall, 600 16th Street, San Francisco, CA 94158, USA; 5 Department of Molecular Biology, Princeton University, 216 Schultz Laboratory, Princeton, NJ 08544, USA; 6 Department of Biological Chemistry, University of California, Los Angeles, 310 BSRB, Los Angeles, CA 90095, USA

## Abstract

**Background:**

Ire1 is a signal transduction protein in the endoplasmic reticulum (ER) membrane that serves to adjust the protein-folding capacity of the ER according to the needs of the cell. Ire1 signals, in a transcriptional program, the unfolded protein response (UPR) via the coordinated action of its protein kinase and RNase domains. In this study, we investigated how the binding of cofactors to the kinase domain of Ire1 modulates its RNase activity.

**Results:**

Our results suggest that the kinase domain of Ire1 initially binds cofactors without activation of the RNase domain. RNase is activated upon a subsequent conformational rearrangement of Ire1 governed by the chemical properties of bound cofactors. The conformational step can be selectively inhibited by chemical perturbations of cofactors. Substitution of a single oxygen atom in the terminal β-phosphate group of a potent cofactor ADP by sulfur results in ADPβS, a cofactor that binds to Ire1 as well as to ADP but does not activate RNase. RNase activity can be rescued by thiophilic metal ions such as Mn^2+ ^and Cd^2+^, revealing a functional metal ion-phosphate interaction which controls the conformation and RNase activity of the Ire1 ADP complex. Mutagenesis of the kinase domain suggests that this rearrangement involves movement of the αC-helix, which is generally conserved among protein kinases. Using X-ray crystallography, we show that oligomerization of Ire1 is sufficient for placing the αC-helix in the active, cofactor-bound-like conformation, even in the absence of cofactors.

**Conclusions:**

Our structural and biochemical evidence converges on a model that the cofactor-induced conformational change in Ire1 is coupled to oligomerization of the receptor, which, in turn, activates RNase. The data reveal that cofactor-Ire1 interactions occur in two independent steps: binding of a cofactor to Ire1 and subsequent rearrangement of Ire1 resulting in its self-association. The pronounced allosteric effect of cofactors on protein-protein interactions involving Ire1's kinase domain suggests that protein kinases and pseudokinases encoded in metazoan genomes may use ATP pocket-binding ligands similarly to exert signaling roles other than phosphoryl transfer.

## Background

The unfolded protein response (UPR) is an intracellular signaling pathway that provides homeostatic feedback regulation between the endoplasmic reticulum (ER) and the gene expression program in the nucleus. To this end, the UPR senses the conditions inside the ER, detecting an imbalance between newly synthesized protein influx and the protein-folding capacity in the ER lumen, and activates a corrective response. When activated, the UPR drives a broad transcriptional program that adjusts the abundance of the ER [1].

The primary signal transduction device in the UPR is an ER membrane-resident sensor of misfolded proteins, Ire1. Ire1 is conserved from yeast to mammalian cells [2-4]. According to one model, unfolded proteins act as ligands that bind directly to the Ire1 luminal domain [5,6]. Another model posits an indirect mode of activation in which the ER-luminal chaperone BiP plays a central role [7]. Both views converge on the concept that oligomerization of the Ire1-luminal domain is crucial for signal propagation across the membrane. Oligomerization of Ire1's luminal domains leads to a local increase in the concentration of Ire1's cytoplasmic kinase and RNase domains, which are tethered to the ER-luminal domain through a transmembrane segment. Recent structural insights suggest that oligomerization activates the cytoplasmic modules by their cooperative assembly into an ordered oligomer [8]. The kinase domains undergo *trans*-autophosphorylation, which installs phosphate-mediated salt bridges between adjacent Ire1 monomers to stabilize the oligomer. The resulting juxtaposition of Ire1's RNase domains assembles and stabilizes the RNase active site [8,9]. Ire1 then initiates the unconventional splicing of *HAC1 *(yeast) or *XBP1 *(metazoan) mRNA by cleaving either mRNA at two conserved sites to excise an intron [8,10]. Transfer RNA ligase completes the mRNA splicing reaction in yeast [4], and an as yet unknown enzyme ligates the exons in metazoans. Intron removal allows the production of the UPR transcription activators Hac1 and XBP1, respectively. The transcription factors upregulate UPR target genes, closing a feedback loop that adjusts the ER's protein-folding capacity according to need.

The kinase domain of Ire1 is structurally and biochemically similar to that of other protein kinases, including cSrc [11], epidermal growth factor receptor (EGFR) [12], PKR [13], GCN2 [14], cyclin-dependent kinase 2 (CDK2) [9], Aurora [15], cAMP [16] and mitogen-activated protein kinase kinase 1 [17] (Additional file 1, Figures S1 and S2). Notably, binding of nucleotides and synthetic ligands to the kinase domain of Ire1 allosterically activates the Ire1 RNase [8,18]. Ire1's RNase activity provides a built-in reporter of kinase conformation, establishing Ire1 as a unique model in which conformational properties of a kinase can be studied separately from the phosphoryl transfer step by using a sensitive and quantitative enzymatic readout.

ADP serves as a cofactor [10] which stimulates RNase activity of *Saccharomyces cerevisiae *Ire1 *in vitro *by approximately 200-fold [8]. It has been proposed that ADP and other ATP pocket ligands stabilize Ire1 in a conformation that forms oligomers [8]. As protein kinases related to Ire1 can assume two globally different conformational states, commonly referred to as "inactive" and "active" [12], Ire1 monomers were thought to exist in an inactive conformation and cofactors were thought to stabilize the active conformation via occupancy of the ATP pocket [18]. This simple model, however, cannot explain the paradoxical observation that ADP has been reported to be a better cofactor than the bulkier ATP [10]. Binding of ATP to the ATP pocket does not fully trigger the RNase-activating conformational change in Ire1, arguing against the model that ATP pocket occupancy is sufficient to lock Ire1 into the active conformation and to activate RNase. In the present study, we carried out a quantitative analysis of Ire1 oligomerization and RNase activation properties with a series of cofactors to resolve this conundrum and to glean insights into the allosteric mechanism by which cofactors control the activity of Ire1 RNase.

## Results

### RNase activity quantitatively correlates with Ire1 oligomerization

**Figure 1:**
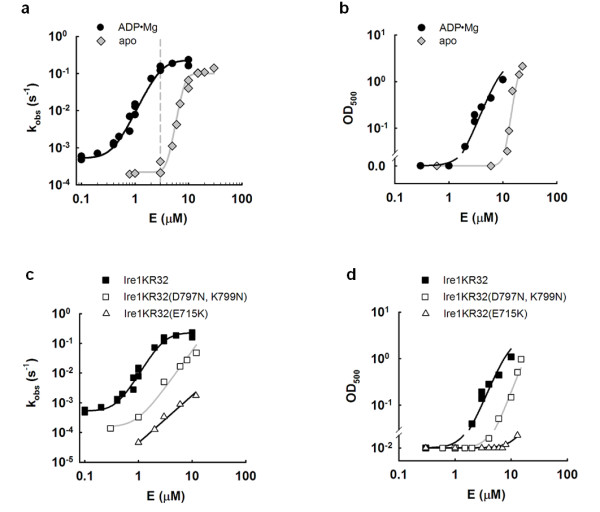
**Linking RNase activity and high-order oligomerization of Ire1 kinase/RNase**. **(a) **Cooperative activation of Ire1 RNase with and without 2 mmol ADP Mg [8]. The dashed line marks the standard assay condition at 3 μmol Ire1KR32. **(b) **Opacity at 500 nm versus concentration of Ire1KR32 with and without 2 mmol ADP Mg. **(c) **RNase activation profiles for Ire1KR32, Ire1KR32(D797N, K799N) bearing a catalytically disabled, unphosphorylated kinase domain, and for Ire1KR32(E715K). Reactions were conducted as in (a) in the presence of 2 mmol ADP Mg. **(d) **OD_500 _response versus concentration of Ire1KR32, Ire1KR32(D797N, K799N) and Ire1KR32(E715K). Assays were conducted as in (b) in the presence of 2 mmol ADP Mg.

We observed previously [8] that Ire1 RNase is activated upon high-order oligomerization of Ire1's kinase/RNase domains (Figure 1a). At sufficiently high concentrations, a cytosolic portion of Ire1 (Ire1KR32) forms large-scale oligomers that scatter visible light and display high RNase activity [8]. Ire1KR32 contains Ire1's cytoplasmic kinase and RNase domains, is autophosphorylated as isolated from the *Escherichia coli *expression host and bears an N-terminal 32-aa extension derived from the linker that connects the Ire1 kinase and transmembrane domains. This N-terminal extension is important for optimal oligomerization and RNase activity of Ire1 [8]. As Ire1KR32 oligomerization resulted in cloudy solutions visible to the naked eye, we explored whether sample transparency could be used to evaluate the oligomer formation quantitatively. We found that turbidity of Ire1KR32 solutions measured as an optical density at 500 nm (OD500) indeed provides a reliable, quantitative metric of oligomerization (Figure 1b).

As a function of enzyme concentration, the OD_500 _of Ire1KR32 solutions increased cooperatively by approximately two orders of magnitude in both the absence and presence of the activating cofactor ADP Mg (Figure 1b, diamonds and circles, respectively). Observed Hill coefficients (*n *= 3 for Ire1 ADP Mg and *n *= 8 for apo-Ire1) were in close agreement with those determined in an enzymatic assay that monitors Ire1's RNase activity (*n *= 3.5 for Ire1 KR32 ADP Mg and *n *= 8 for apo-Ire1KR32 [8]) (Figure 1a). In the presence of the ADP Mg cofactor, both OD_500 _and RNase profiles shift similarly toward lower Ire1KR32 concentrations, which, together with similar Hill coefficients, indicates that both assays monitor the same physical phenomenon: high-order Ire1 oligomerization.

As shown in an overlay of the OD_500 _and RNase measurements (Additional file 1, Figure S3), both assays are indeed superimposable. OD_500 _was below the detection limit at Ire1KR32 concentrations that already displayed activated RNase, indicating that active Ire1KR32 oligomers at these concentrations are too sparse to result in detectable turbidity. By contrast, OD_500 _values continued to rise after RNase activity reached a kinetic plateau due to the onset of a saturating regime *k*_2_, as the oligomer concentration exceeded the *K*_m _for RNA binding [19]. The RNase and OD_500 _readouts therefore complement one another and allow monitoring of the log-linear oligomerization over a broader range of Ire1 concentrations than either assay in isolation. The RNase activity provides a more sensitive measurement and detects oligomers at concentrations several orders of magnitude smaller than those detectable by OD_500 _readings.

Recombinantly expressed Ire1KR32 used in these studies contains approximately 20 phosphates [8]. We showed previously that removal of these phosphates by mutagenesis of the kinase active site shifted the activation profile to higher Ire1KR32 concentrations [8]. To further verify that the RNase-based readout of oligomerization and the more direct readout based on OD_500 _absorbance are in agreement, we measured the concentration dependence of an unphosphorylated variant of Ire1KR32, Ire1KR32(D797N, K799N). This double-mutant carries mutations of residues that are required for γ-phosphate coordination and catalysis of phosphotransfer but otherwise do not disrupt nucleotide coordination at the ATP pocket. Recombinantly expressed Ire1KR32(D797N, K799N) is entirely unphosphorylated as established by mass-spectrometry, and phosphotransfer-inactive [20]. As shown in Figures 1c and 1d, both RNase activity and OD_500 _profiles were shifted to higher enzyme concentrations for Ire1KR32(D797N, K799N). Note that the data include the characterization of an additional Ire1 mutant Ire1KR32(E715K), which also exhibited a consistent shift in both assays and is discussed below.

Therefore, both RNase and OD_500 _readouts report robustly on cofactor addition (Figures 1a and 1b) and perturbations due to mutations in Ire1 (Figures 1c and 1d). We show below that the equivalency between the optical and enzymatic readouts also extends to all other variables tested, such as chemical substitution in the ADP cofactor and addition of thiophilic metal ions in the presence of an ADP isostere, ADPβS. The data in Figure 1 therefore indicate that RNase activity and OD_500 _provide reliable readouts for Ire1KR32 oligomerization and support the model [8] in which oligomerization and RNase activation are mechanistically coupled.

### Activation of Ire1 RNase by different cofactors

**Figure 2:**
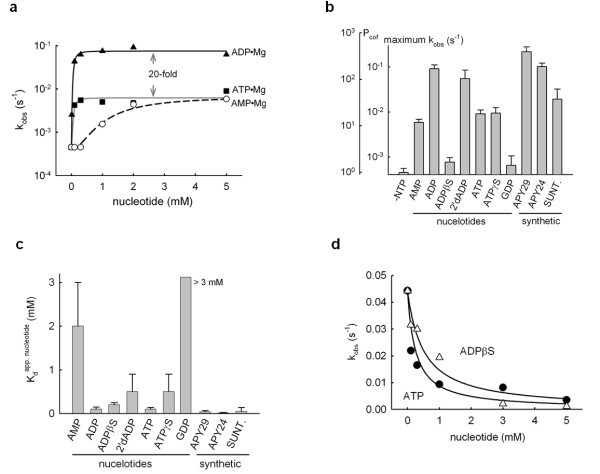
**Allosteric activation of Ire1 RNase by ligands binding to the kinase domain**. **(a) **Activation of Ire1KR32 by AMP, ADP and ATP. The *k*_obs _values of RNase activity were determined as a function of nucleotide Mg^2+ ^concentration (see Methods). **(b) **and **(c) **Characterization of Ire1 activity with a panel of nucleotides and synthetic ligands. Maximum Ire1 RNase stimulation achieved with cofactors at saturation **(b) **and apparent cofactor binding constants **(c) **are shown. The scale of cofactor potency (*P*_cof_) is indicated in **(b)**. SUNT = sunitinib. Cofactor structures are shown in Additional file 1, Figure S4. **(d) **Measurement of binding constants for ADPβS and ATP via inhibition of a reaction stimulated with 0.2 mmol ADP. *K*_i _values of 0.20 ± 0.07 mmol and 0.10 ± 0.04 mmol were determined for ADPβS and ATP, respectively. The data were fit to the binding isotherm and corrected for the 0.2 mmol ADP background. All reactions contained 3 μmol Ire1KR32 and 2 mmol Mg^2+^. Error bars show standard errors of binding isotherm fitting.

To quantitatively define the potency of different Ire1 cofactors, we measured the activation of Ire1KR32 RNase *in vitro *following the site-specific cleavage of a ^32^P-labeled RNA hairpin substrate. We found that at saturating concentrations, nucleotides AMP and ATP both activated Ire1KR32 by approximately tenfold (Figures 2a and 2b). Notably, although the effects at saturation were similar, the apparent affinity for AMP was tenfold lower than that observed for ATP (Figure 2c). By contrast, ADP bound with an apparent affinity similar to that of ATP, but ADP binding resulted in 20-fold stronger activation of the enzyme (Figures 2b and 2c). Among other nucleotides tested, only 2'-deoxy(ADP) stimulated Ire1KR32 RNase comparably to ADP (Figure 2b), although it bound more weakly (Figure 2c). The divergence of the apparent cofactor binding constants and maximal stimulation of RNase activity at saturating cofactor concentrations extended to other cofactors (Figures 2b and 2c). Thus, surprisingly, cofactor binding and cofactor-induced conformational changes that promote Ire1 oligomerization can be experimentally uncoupled. As binding of the larger ATP molecule fails to activate Ire1KR32 to the same extent as does binding of ADP, resulting in an order-of-magnitude smaller response (Figure 2a), we concluded that mere occupancy of the ATP pocket does not effect optimal Ire1KR32 activation.

To describe different cofactors quantitatively, we defined a "potency of cofactor" (*P*_cof_)

as the ratio of the *k*_obs _of Ire1 with saturating amounts of cofactor bound over the *k*_obs _of apo-Ire1, both measured under standard assay conditions, which we defined as 3 μmol Ire1KR32. At this Ire1KR32 concentration, maximum fold stimulation by ADP was achieved (Figure 1a, dashed line; see also [8]). The relationship between the macroscopic conformational parameter *P*_cof _and the microscopic equilibria involved in oligomerization is described in the Discussion section and in greater detail in Additional file 1, Supplementary Analysis 1.

When cofactors are compared using the *P*_cof _metric, the RNase activity of apo-Ire1 defines the baseline with *P*_cof _= 1. The presence of bound ADP stimulates Ire1KR32 strongly with *P*_cof _approximately 200), whereas the presence of bound AMP or ATP is less effective (*P*_cof _approximately 10) (Figure 2b). Similarly, synthetic small molecules that bind to the nucleotide-binding pocket in Ire1 (Additional file 1, Figure S4) can be described by *P*_cof _(Figures 2b and 2c). Sunitinib, an ATP-mimicking drug developed as a competitive inhibitor of EGFR kinase, activated Ire1KR32 to a similar extent as AMP and ATP (*P*_cof _approximately 30), whereas APY24 and APY29 activated Ire1 as well as or better than ADP (*P*_cof _approximately 200 to 300) (Figure 2b) [8].

### A β-phosphate-coordinated metal ion facilitates Ire1 oligomerization

**Figure 3:**
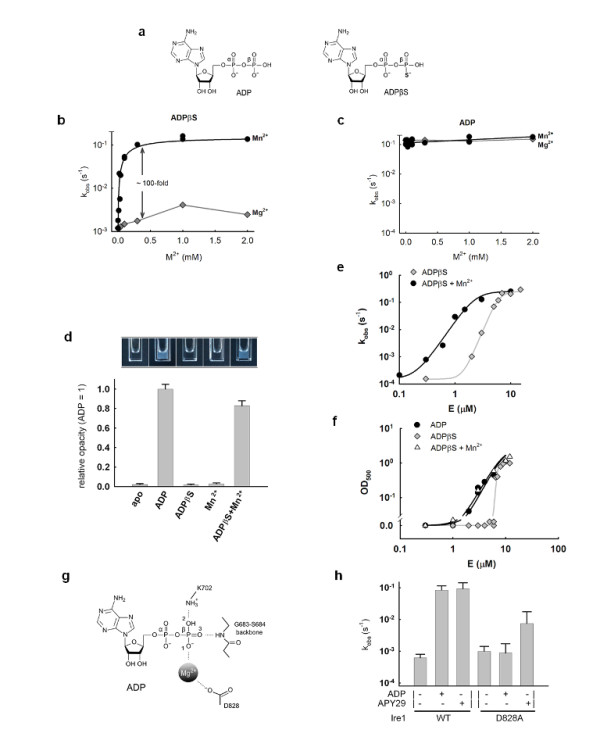
**Manganese rescue of Ire1 RNase activity and oligomerization in the presence of ADPβS**. **(a) **Structures of ADP and ADPβS. **(b) **and **(c) **The effect of increasing Mg^2+ ^and Mn^2+ ^concentrations on Ire1 RNase activity in the presence of 2 mmol ADPβS **(b) **or 2 mmol ADP **(c)**. The buffer contained 0.2 mmol Mg^2+^. All reactions contained 3 μmol Ire1KR32. Assays were conducted as described in Figure 1(a)a. **(d) **Visible oligomerization of Ire1KR32 in the absence of cofactors and in the presence of 1 mmol ADP, 1 mmol ADPβS, 1 mmol Mn^2+ ^and 1 mmol ADPβS + 1 mmol Mn^2+^. Assays contained 10 μmol Ire1KR32 and were conducted as described in Figure 1(b). All reactions contained 2 mmol Mg^2+^. The bar chart below the image shows relative opacity of each sample. **(e) **Cooperative activation of Ire1KR32 RNase assayed using ^32^P-5'-HP21 substrate as described in Figure 1(a). Reactions contained 2 mmol ADPβS, 2 mmol Mg^2+ ^and either 0 or 2 mmol Mn^2+ ^as indicated. **(f) **OD_500 _versus concentration of Ire1KR32 in the presence of 2 mmol ADP, 2 mmol ADPβS and 2 mmol ADPβS + 2 mmol Mn^2+^. All reactions contained 2 mmol Mg^2+^. **(g) **Interactions involving the β-phosphate in the Ire1 ADP Mg complex (PDB ID 2rio). Note that all three nonbridging oxygen atoms of the β-phosphate form contacts to either protein residues or Mg^2+^. **(h) **The rate constants for Ire1 and Ire1(D828A) in the presence of ADP Mg^2+ ^and APY29. Error bars show standard errors of single-exponential fitting of corresponding time courses.

Strikingly, the close ADP-mimic ADPβS did not activate Ire1KR32 (Figure 2b; *P*_cof _approximately 1; ADPβS contains a sulfur atom that replaces a nonbridging oxygen atom on the β-phosphate, Figure 3a). This finding was paradoxical because ADPβS bound to Ire1 with the same affinity as ADP and ATP, as indicated by its apparent *K*_i _in a competitive inhibition assay of Ire1KR32's ADP-stimulated RNase (Figure 2d) and by measurements of the small RNase stimulation by ADPβS in the absence of ADP (Figure 2c). Thus we conclude that the ATP pocket can be occupied by a tightly binding cofactor without activating RNase.

The sulfur substitution in ADPβS introduces a "soft" atom, which has a larger covalent radius than that of oxygen. Sulfur does not form strong hydrogen bonds and does not coordinate strongly with "hard" cations such as magnesium [21,22]. Elegant "metal specificity switch and rescue" experiments that are well established for ribozymes showed that metal ion coordination disrupted as a result of oxygen-sulfur substitutions can sometimes be restored if a softer metal cation such as Mn^2+ ^is supplied to the reaction [21,22]. Indeed, when we added Mn^2+ ^to the assay buffer, we observed complete restoration of ADPβS cofactor activity to the level of ADP Mg (Figure 3b; *P*_cof _approximately 100).

The rescue was specific for a sulfur-soft metal ion pair because ADPβS did not activate Ire1 by more than a few fold in the presence of Mg^2+ ^(Figure 3b) and because Cd^2+^, another thiophilic metal, also rescued the activity (Additional file 1, Figure S5; *P*_cof _approximately 30). Furthermore, neither Mn^2+ ^nor Cd^2+ ^stimulated reactions with oxo-ADP (Figure 3c; Additional file 1, Figure S5). These data strongly point to a functional connectivity between the β-phosphate of ADP, Mg^2+ ^ion coordination and a conformational change in the Ire1 kinase domain, resulting in activation of Ire1 RNase.

By contrast, such connectivity was not observed for the γ-phosphate of ATP, which also contacts the Mg^2+ ^ion in crystal structures of protein kinases. ATPγS bound to Ire1 slightly more weakly than ATP (Figure 2c), but at saturation it functioned indistinguishably from ATP (Figure 2b; *P*_cof _approximately 10 for ATP and ATPγS).

As expected, RNase activation by ADPβS and Mn^2+ ^fully coincided with Ire1KR32 oligomerization monitored as OD_500_. Samples of Ire1KR32 were transparent in the absence of cofactors or in the presence of ADPβS or Mn^2+ ^alone. By contrast, white oligomer solutions formed in the presence of ADP + Mg^2+ ^or ADPβS + Mn^2+ ^(Figure 3d; 2 mmol magnesium background was present in all reactions shown). Measuring RNase activity and OD_500 _as a function of Ire1KR32 concentration further confirmed that ADPβS-saturated Ire1KR32 behaved similarly to apo-Ire1KR32. Upon addition of Mn^2+^, cooperative activation profiles with ADPβS became indistinguishable from those obtained with Ire1KR32 and oxo-ADP Mg (Figures 3e and 3f).

Because the β-phosphate can rotate around the α-β bridging bond, replacement of one β-oxygen atom by sulfur leaves two more oxygen atoms on the β-phosphate available for coordination of Mg^2+^. The deleterious effect of the oxygen-to-sulfur substitution in ADPβS therefore implies that all three nonbridging oxygen atoms on the β-phosphate form functionally important interactions, all of which are required for the conformational change in Ire1's kinase domain. Thus, every nonbridging oxygen atom must obligatorily either coordinate Mg^2+ ^or form hydrogen-bonding contacts to the protein. Detrimental effects of the steric bulk introduced by the larger sulfur atom can be disregarded, because ADPβS works as well as ADP in the presence of Mn^2+^.

In the crystal structure of the Ire1 ADP complex (PDB ID 2rio) [9], nonbridging oxygen 1 (Figure 3g) coordinates to Mg^2+^, nonbridging oxygen 2 forms a hydrogen bond through conserved lysine K702 and nonbridging oxygen 3 forms a hydrogen bond to the backbone of strand β1 (GXGXXG motif). This network of connectivity is conserved among protein kinases [23], suggesting that lessons learned from the nucleotide effects on Ire1 may be broadly applicable to other protein kinases.

In Ire1, the β-phosphate-coordinating Mg^2+ ^ion additionally coordinates to the conserved aspartate D828, which is functionally important for Ire1 RNase activation (Figures 3g and 3h). As shown in Figure 3h, removal of this interaction via a D828A mutation in Ire1KR32 results in a loss of Ire1 responsiveness to ADP Mg, consistent with the importance of the magnesium coordination demonstrated by the studies of ADPβS above. Addition of the magnesium-independent cofactor APY29 [8] (Figure 2b and Additional file 1, Figure S4), which replaces the β-phosphate-magnesium moiety with an aromatic ring, partially activated Ire1KR32(D828A) (*P*_cof _approximately 10; Figure 3h). Therefore, the Ire1KR32(D828A) mutant retains the intrinsic ability to become activated by cofactors, and the loss of responsiveness to ADP Mg^2+ ^arises, at least partially, from the impaired magnesium coordination.

On the basis of the data presented so far, we conclude that the β-phosphate-interactions of ADP with Ire1 kinase form a "β-phosphate latch". This latch involves a centrally positioned Mg^2+ ^ion that lies at the core of the molecular switch that controls Ire1 oligomerization and thereby its RNase activity. For natural adenosine nucleotides, this latch is engaged via a highly specific hydrogen bonding and metal coordination network. For synthetic ligands, such as APY29, the β-phosphate latch is bypassed by metal-independent interactions such as hydrophobic and/or hydrogen-bonding contacts.

### The position of the β-phosphate is dynamically linked to the conformation of the kinase domain

**Figure 4:**
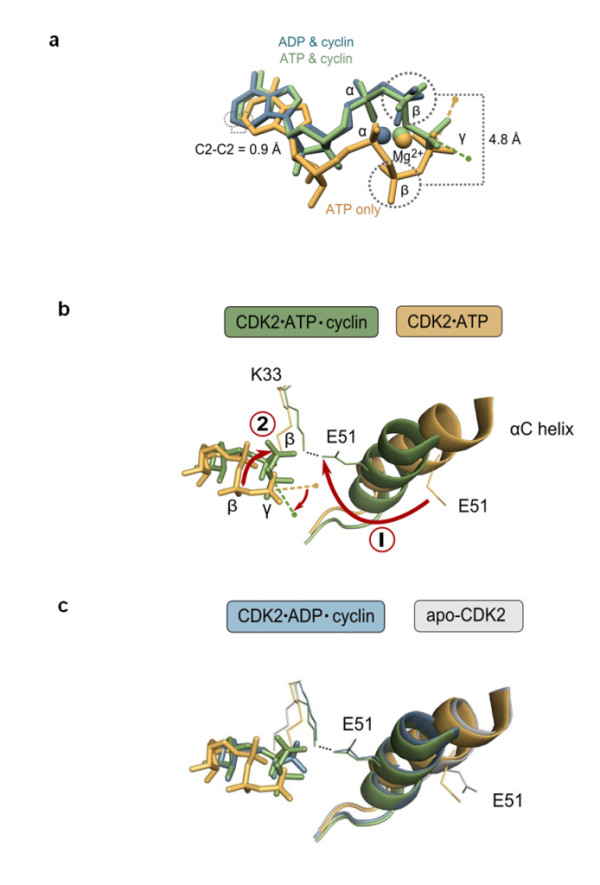
**Coupled motion of αC-helix and β-phosphate in an Ire1-like kinase CDK2**. **(a) **ATP bound to CDK2 (orange trace; CDK2 is in the inactive conformation) is, for most of its atoms, well aligned with ADP and ATP bound to a CDK2 cyclin complex (blue and green traces, respectively; CDK2 is in the active conformation), except for the β-phosphate, which is displaced by approximately 4.8 Å. **(b) **Key conformational rearrangements during inactive ↔ active transition in CDK2. Movement of αC-helix and E51 (1) is coupled to the movement of β-phosphate (2). The conformational change involves formation of a salt bridge between E51 and K33, formation of an interaction between K33 and β-phosphate and rotation of the γ-phosphate for in-line attack by the OH group of the substrate (small arrow connecting dashed lines indicates change in geometry) [41]. Coordinates are from PDB ID: 1hcl (apo-CDK2), 1b39 (ATP-bound CDK2), 1qmz (ATP-bound CDK2 with cyclin) and 1gy3 (ADP-bound CDK2 with cyclin). **(c) **The conformation of the CDK2 kinase domain in the ATP-bound state (inactive conformation with no cyclin bound) is similar to the apo-state (inactive conformation with no cyclin bound) rather than to the ADP-bound state (active conformation with cyclin bound). A corresponding comparison of several additional kinases is shown in Additional file 1, Figure S1.

Equivalently placed cofactor-bound Mg^2+ ^ions are found in other protein kinases that are structurally similar to Ire1 [9,11-17], including the closely related and structurally well-characterized cell cycle regulator kinase CDK2. The high structural similarity between CDK2 and Ire1 kinase domains (Additional file 1, Figure S2) suggests that insights gained from CDK2 may help increase the understanding of Ire1 function and *vice versa*. Comparisons of aligned structures of CDK2 ADP Mg, CDK2 ATP Mg with and without bound cyclin (a CDK2 activator) and apo-CDK2 reveal only a minor displacement (root mean square displacement approximately 1 Å) of the position of the adenosine base (Figure 4a), yet phosphate atoms of the bound nucleotides move much larger distances. The β-phosphate in particular stands out as the most rearranged part of the nucleotides, occupying one of two positions that are 4.8 Å apart. As we discuss below, these two states of the nucleotide and the kinase domain are linked to the rearrangement of the αC-helix common to protein kinases and are indicative of switching CDK2 between its inactive (αC-helix "out", E51 points outside the ATP pocket) and active (αC-helix "in", E51 points inside the ATP pocket) conformations (Figure 4b) [12]. The same connection between the β-phosphate position and the kinase conformation could be established for a well-characterized tyrosine kinase cSrc that also has been crystallized in several conformations (Additional file 1, Figure S1a).

Two relevant conclusions arise from published crystal structures of protein kinases. First, ATP can bind to a kinase domain without noticeably altering its inactive conformation, as judged by the unchanged position of the αC-helix in apo-CDK2 and in CDK2 ATP Mg complexes (Figures 4b and 4c). Therefore, in agreement with our studies on Ire1KR32 and ADPβS, conformational changes in the kinase domain do not necessarily occur in response to occupancy of the nucleotide binding pocket. Second, transition from the inactive to the active conformation upon the binding of cyclin to CDK2 ATP results in a pronounced (approximately 10 Å) movement of the αC-helix (Figure 4b, 1), demarcating its switch to the active conformation [12], and in an approximately 5-Å movement of the β-phosphate (Figure 4b, 2). This global conformational rearrangement positions a conserved glutamate E51^CDK2 ^to interact through a salt bridge with the conserved lysine K33^CDK2^, which docks to the β-phosphate. The docking of the β-phosphate aligns the γ-phosphate in the proper geometry for phosphotransfer [24] (Figure 4b, 2).

Our experiments demonstrating the rescue of Ire1KR32 ADPβS activity by Mn^2+ ^suggest that β-phosphate insertion does not occur merely in response to a conformational change in the kinase domain. Instead, it supplies appreciable free energy for stabilizing Ire1 kinase and, by extension, other related protein kinases in the active conformation with the αC-helix in the "in" orientation.

The movement involving αC-helix is a dynamic feature of many protein kinases [12,14,25]. Important to the work presented here is that the residues for β-phosphate placement in CDK2 (E51 and K33) are conserved in Ire1 (E715 and K702, respectively). To assess experimentally whether the αC-helix in Ire1 functions in the conformational switch, we probed the predicted salt bridge K702-E715 by mutagenesis. Because K702 is also directly involved in cofactor binding (Figure 4b), we judged that the effects of any K702 mutations could not be interpreted straightforwardly and therefore focused the mutagenesis on E715. We chose to mutate E715 to lysine, creating Ire1KR32(E715K), to minimize perturbations to space filling, local hydrophobicity and H-bonding capability. The E715K mutation introduces a repulsive electrostatic interaction with K702 and is expected to destabilize the αC-helix "in" conformation.

In agreement with this expectation, the Ire1KR32(E715K) mutant displayed a vastly decreased RNase activity (Figure 1c, triangles) and did not oligomerize at concentrations at which wild-type Ire1KR32 produced large amounts of oligomers (Figure 1d, triangles versus squares). As the mutational perturbation of αC-helix was expected to affect the phosphotransfer activity of the Ire1 kinase, it was important to rule out that the oligomerization defect of the Ire1KR32(E715K) mutant and its concomitant loss of RNase activity did not result solely from a loss of phosphorylation. The effect of the loss of phosphorylation could be evaluated readily by comparing Ire1KR32(E715K) with Ire1KR32, which would be entirely unphosphorylated. To this end, we used the double-mutant Ire1KR32(D797N, K799N), which has a catalytically inactive kinase domain, purifies with no phosphates attached and retains readily detectable RNase activity [20]. At concentrations that allowed Ire1KR32(D797N, K799N) to oligomerize nearly to the level of wild-type Ire1KR32 and to exhibit nearly maximal RNase activity, Ire1KR32(E715K) showed only trace RNase activity and only trace levels of oligomers in the OD_500 _readout (Figures 1c and 1d). These findings confirm that the deleterious effects of the E715K mutation cannot be accounted for even by the complete loss of phosphorylation and therefore support the model that E715 controls the conformational properties of Ire1.

This model is also supported by the recent crystal structure of unphosphorylated human Ire1α, which shows human Ire1α in an inactive conformation. In this structure, the β-phosphate latch is disengaged, the activation loop and the αC-helix are in the inactive conformation and the salt bridge K599-E612 (corresponding to K702-E715 in yeast Ire1) is disrupted [26]. While formally remaining conjecture, it is very likely that the K599-E612 interaction and the β-phosphate insertion also occur in the active conformation of human Ire1α, because the active conformations are essentially the same for all protein kinases [27]. According to this view, the functional analyses of Ire1KR32(E715K) presented herein support the conformational similarity of Ire1, CDK2, cSrc, EGFR and related protein kinases inferred from their sequence and structural similarity.

### Crystal structure of an Ire1KR32 oligomer with an apo-kinase domain

The results presented so far suggest a model in which ADP Mg locks Ire1 into the αC-helix "in" conformation, which favors oligomerization and thereby activates RNase. From the principle of coupled equilibria, it follows that oligomerization, if it can be induced without kinase ligand binding, would likewise lock the kinase domain into the active αC-helix "in" conformation. Indeed, high concentrations of apo-Ire1 induce cooperative oligomerization and activation of RNase, illustrating the ability of apo-Ire1 to oligomerize without cofactors (Figures 1a and 1b; see also [8]). Structural insights into this apo-Ire1 oligomer, however, have been missing because to date attempts to crystallize the oligomeric Ire1KR32 in the absence of ATP pocket-binding cofactors have not been successful. In the present study, we circumvented this problem by introducing a new ligand, an oligonucleotide bound to the RNase domain, that resulted in diffracting crystals of IreKR32 in its oligomeric state but with a ligand-free apo kinase domain.

**Figure 5:**
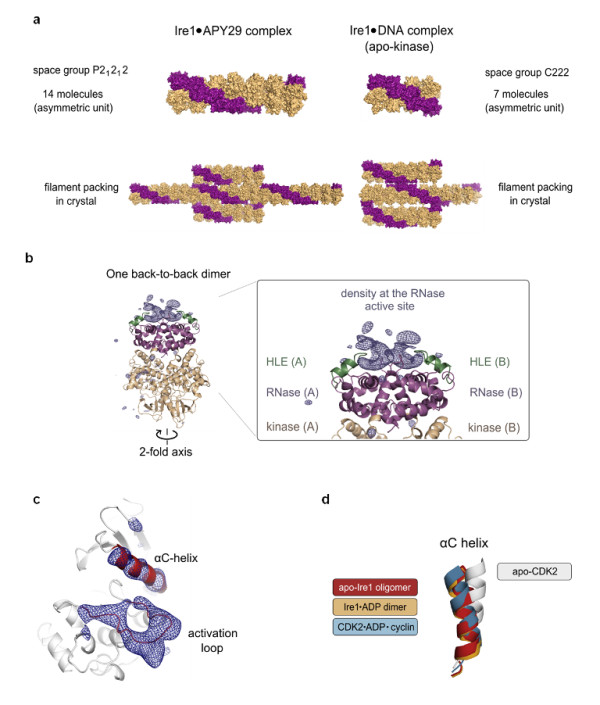
**Crystal structure of the Ire1KR32 oligomer obtained without ligands in the ATP pocket**. **(a) **Ire1 kinase/RNase forms an oligomer with the synthetic ligand APY29 (crystal P2_1_2_1_2) and with a 6-mer DNA oligonucleotide that served as a noncleavable mimic of Ire1's RNA substrate (crystal C222). The sequence dCdCdGdCdAdG of the oligonucleotide is derived from the splice site of *HAC1 *mRNA. The filaments in the P2_1_2_1_2 and in the C222 structures pack using entirely different sets of crystal contacts. **(b) **Simulated annealing (2,000 K) NCS-averaged *F*_obs _- *F*_calc _difference map of the twofold symmetric density in the RNase active site (contoured at 5.5 σ). **(c) **Simulated annealing (2,000 K) NCS-averaged F_obs _- F_calc _omit map for the αC-helix (contoured at 5.5 σ) and for the activation loop (contoured at 4.5 σ) in the C222 structure (red). Omit maps were calculated with αC-helix and activation loop residues deleted from all seven monomers prior to refinement. Analogous maps calculated by simulated annealing without NCS restraints are shown in Additional file 1, Figure S7. **(d) **Superposition of CDK2 and Ire1KR32 in the apo-bound state (this work) and the ADP-bound state (PDB ID 2rio) shows that the αC-helix of apo-Ire1KR32 (in the C222 structure) occupies the same conformation as in Ire1 ADP and in CDK2 ADP cyclin complexes, which is distinct from that in the inactive apo-CDK2 structure.

To this end, we used a splice-site mimic oligonucleotide (CCGCAG) containing 2'-deoxy substitutions throughout to prevent its degradation by Ire1. Oligonucleotide-complexed Ire1KR32 crystallized in space group C222 and diffracted to 6.0 to 6.6 Å (Table 1 and Additional file 1, Figure S6). The asymmetric unit contained seven Ire1KR32 molecules. The asymmetric units packed to form the same oligomeric structure as in the 3.2-Å complex previously crystallized with the small-molecule activator APY29 in the kinase active site, which crystallized in space group P2_1_2_1_2 and had 14 Ire1KR32 molecules per asymmetric unit (PDB ID 3fbv) (Figure 5a). The oligomers in C222 and P2_1_2_1_2 structures pack via a different set of crystallographic contacts (Figure 5a, bottom; note the presence of RNase/RNase interfilament contacts in the C222 structure but not in the P2_1_2_1_2 structure), strongly supporting the notion that the Ire1KR32 oligomer is a stable assembly not resulting from a particular crystal environment. Electron density for the bound substrate was observed in the RNase active site (Figure 5b). Its implications for the catalysis of RNA cleavage are discussed in a separate publication [19].

**Table 1:** Data collection and refinement statisticsa

Data collection and refinement	Statistics	Subcategory	Value
Data collection	Cell dimensions	Space group	C222
		a, b, c (Å)	91.67, 580.82, 177.99
		α, β, γ (°)	90, 90, 90
		Resolution (Å)	97-6.60 (6.8-6.60)
		I/σ(I)	16.62 (1.09)
		R_pim_^b ^(%)	4.4 (88.3)
		Number of reflections	9402 (766)
		Completeness (%)	99.9 (99.9)
Data refinement	Number of atoms (in one monomer)	Protein	3399
		Resolution (Å)	97-6.60
	Rigid body (with NCS)	Angles (^o^)	0.925
		Bonds (Å)	0.006
		R_work_/R_free_	0.2863/0.3170
	Simulated annealing (with NCS)	t (°K)	1,000
		Angles (°)	1.197
		Bonds (Å)	0.007
		R_work_/R_free_	0.2731/0.3451

^a^Refinement was done using rigid body protocol. Simulated annealing was not used in final analysis, except for calculations of omit maps. Values for the highest resolution shell are shown in parentheses. ^b^Precision-indicating merging R-factor [42].

Definitions: NCS - non-crystallographic symmetry; R_pim _- precision-indicating merging factor; R_work _- crystallographic residual factor; R_free _- free crystallographic residual factor.

Although at 6.6-Å resolution it is not possible to identify positions of individual side chains, secondary structural elements can be resolved, as evidenced by low-resolution crystal structures of other macromolecules [28], especially if a high-resolution structure can be used for phasing. Sevenfold noncrystallographic symmetry averaging greatly enhanced the effective quality of the Ire1-oligonucleotide complex density map (Figures 5c versus Additional file 1, Figure S7). A cross-validated SigmaA test [29] showed that useful and statistically significant diffraction spans to approximately 6.0 Å. The simulated-annealing omit electron density maps, in which the model bias for the αC-helix and the activation loop regions is minimized, demonstrate unambiguously that the αC-helix and the activation loop, the two most dynamic parts of kinases, are in the same position as they were in two previous structures, in which either ADP Mg or the synthetic activator APY29 was bound (PDB ID 2rio[9] and PDB ID 3fbv[8]) (Figures 5c and 5d and Additional file 1, Figure S7). These observations are in agreement with the model that oligomerization of apo-Ire1KR32 requires the αC-helix to be in the "in" position. On the basis of these findings, it is likely that oligomerization plays the same role in controlling Ire1 conformation as cyclin binding does in CDK2 and dimerization does in EGFR [12], such that binding of a protein partner stabilizes the αC-helix in the "in" position, even when the kinase active site is empty.

## Discussion

Protein kinases are dynamic enzymes that couple catalysis to pronounced conformational changes. Studies of their dynamics are limited by the intrinsic difficulty of uncoupling enzymatic phosphoryl transfer from conformational properties. In the present study, we exploited the unique facets of Ire1 biology to overcome this shortcoming. The approach was made possible by Ire1's unique property to form easily quantified high-order homo-oligomers upon activation and by Ire1's RNase domain, which provides a natural built-in reporter of the kinase domain conformation. Conformational changes in Ire1's kinase domain modulate the enzyme's predisposition to form oligomers, in which Ire1-Ire1 contacts across multiple interfaces activate Ire1's RNase activity.

The propensity of Ire1 for activation is modulated by three parameters: (1) its local concentration, (2) its phosphorylation state and (3) its interactions with cofactors in the ATP binding site of Ire1's kinase module. In its physiological setting, the local concentration of the cytoplasmic Ire1 kinase/RNase module is responsive to the protein-folding conditions inside the ER lumen. As unfolded proteins accumulate there, the Ire1's luminal domain oligomerizes, thereby concentrating the covalently tethered kinase/RNase domains on the other side of the membrane. This concentration event is thought to provide the primary switch leading to Ire1 activation. In this view, *trans*-autophosphorylation would ensue following juxtaposition of the kinase domains. The phosphorylation state of Ire1 is a dynamic parameter that changes over time and is thought to contribute to a molecular timer. Initial phosphorylation events on the activation loop lead to phosphorylated side chains that form stabilizing salt bridges to neighboring Ire1 molecules in the active oligomer [8], whereas subsequent hyperphosphorylation events appear to promote Ire1 turnoff, perhaps because of oligomer-destabilizing charge repulsion effects [20].

Cofactor binding to the ATP pocket of the kinase domain is expected to provide another regulatory input that strongly modulates the sensitivity of the switch to integrate UPR signaling with the physiological state in the cell's cytoplasm. In particular, it is intriguing to speculate that UPR may be modulated by the ADP/(ATP+AMP) ratio due to the 20-fold higher *P*_cof _of ADP compared to ATP and AMP. Up to an order of magnitude change in the ADP/ATP ratio has been reported during glucose starvation in pancreatic β cells and during apoptosis [30,31]. If this were also the case during the UPR, an increase in the relative ADP level would sensitize Ire1, requiring lower concentrations of unfolded proteins to activate the UPR. An alternative possibility could be that an unknown stress-induced small-molecule metabolite with high *P*_cof _and low *K*_cof_, other than nucleotides, could serve biological roles in modulating Ire1 activity via binding in the ATP pocket. Work in mammalian systems suggests that Ire1 can be activated independently of an accumulation of unfolded proteins in the ER lumen, perhaps by utilizing such a mechanism to shift the oligomerization activation threshold [32]. It has been shown that, in addition to the ATP pocket, Ire1 has a different pocket in the RNase domain and that binding of quercetin to this pocket stimulates Ire1 RNase [33]. This finding opens up a possibility that, in addition to the kinase-binding ligands, ligands binding to the RNase domain of Ire1 contribute to modulation of Ire1 signaling.

Phosphorylation of Ire1 considerably affects its responsiveness to cofactors. In the presence of ADP, fully phosphorylated Ire1KR32 exhibits a 40-fold stronger *P*_cof _(approximately 200) compared to Ire1KR32(D797N, K799N) lacking phosphates (*P*_cof _approximately 5) (data not shown). Therefore, phosphorylation not only promotes Ire1 oligomerization but also "primes" the receptor for sensing ATP pocket-binding cofactors. Notably, unlike P_cof_, binding of cofactors to Ire1 does not appear to depend on Ire1 phosphorylation, as both Ire1KR32 and Ire1KR32(D797N, K799N) exhibit similar *K*_cof _values with ADP (data not shown). The effect of phosphorylation on Ire1 sensitivity to cofactors has also been observed during the study of the quercetin pocket [33]. These observations suggest that modulation of Ire1 signaling by cofactors may be physiologically most important after the UPR has been initiated, amplifying the activity of Ire1 molecules that have already been phosphorylated.

**Figure 6:**
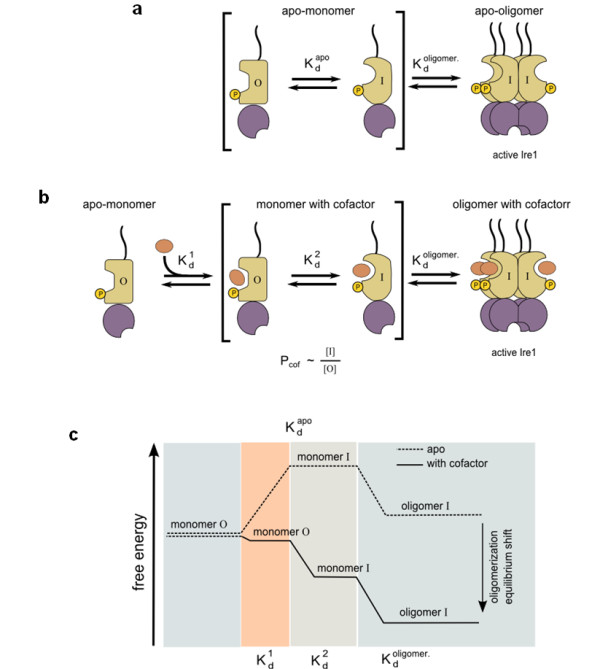
**Model of cofactor action during Ire1 oligomerization and activation**. **(a) **Two-step model for oligomerization and activation of apo-Ire1. **(b) **Three-step model for cofactor-stimulated Ire1 activation involving equilibrium of two distinct cofactor-bound Ire1 species. The macroscopic parameter *P*_cof _depends on the ratio of cofactor-bound Ire1 populations in "inactive" conformation (marked as "O", which designates the αC-helix in the "out" position seen in the inactive conformation of CDK2) and in "active" conformation required for the oligomer assembly (marked as "I", which designates the αC-helix in the "in" position seen in the active conformation of CDK2). The macroscopic apparent cofactor binding constant *K*_cof _depends on the elementary constants  and , whereas the cofactor potency *P*_cof _depends on the  and  (Additional file 1, Supplementary Analysis 1). **(c) **Schematic free-energy diagram for the cofactor-independent and the cofactor-facilitated Ire1 oligomerization that corresponds to the schemes shown in (a) and (b). The shift in the oligomerization equilibrium arises from the stabilization of monomer "I" by cofactors.

By measuring the Ire1 RNase activity in response to kinase-bound small molecules, we resolved two independent steps in cofactor-Ire1 interactions: cofactor binding and the conformational response to a bound cofactor. Noting the wide spectrum of potencies of different cofactors at saturation, P_cof_, we propose a simple model in which cofactors bound in the ATP pocket shift an equilibrium between an inactive and an active conformation of Ire1 (Figure 6). In the model, the inactive conformation "O" corresponds to that of a free Ire1 monomer that has the αC-helix in the inactive, "out" position also observed for other protein kinases. The active conformation "I" corresponds to that of Ire1 oligomers with the αC-helix in the active, "in" position. The conformational equilibrium between the "O" and "I" states depends strongly on the chemical nature of the bound cofactor. Whereas some cofactors, such as ADPβS, bind without noticeably perturbing this equilibrium, other natural and synthetic cofactors effect an equilibrium shift of over two orders of magnitude. The effect of the cofactors on the oligomerization equilibria (Figures 6a versus 6b) is summarized in the free energy diagram shown in Figure 6c. According to our model shown in Figure 6c, cofactors lower the free energy of the "monomer I" state (compare the "monomer I" states on the solid and the dashed lines), ultimately resulting in an equilibrium shift toward oligomerization (compare the "oligomer I" states on the solid and the dashed lines).

Quantitative manifestations of the two steps, apparent cofactor affinity *K*_cof _and cofactor potency *P*_cof_, do not correlate (Figure 2b vs. Figure 2c), suggesting that they arise from distinct (perhaps partially overlapping) subsets of molecular interactions. The ADP/ADPβS comparison (Figure 2) indicates that the kinase hinge-contacting face [8] of the cofactors serves for binding, whereas the K702/E715-contacting face (Figure 4b and Additional file 1 Figure S4) modulates the kinase conformation and could be exploited for designing potent synthetic Ire1 activators. Our findings show that Ire1, as already known for other protein kinases, can exist with the ATP pocket filled by cofactors while remaining in the inactive conformation. With certain cofactors such as ADPβS, this functional state could dominate in solution, a notion previously unappreciated in studies of Ire1.

The macroscopically measured parameters *K*_cof _and *P*_cof _are composite derivatives of the microscopic constants ,  and . (Figure 6 and Additional file 1, Supplementary Analysis 1). The *K*_cof _value depends largely on (and is equal to, when  is large) the true microscopic binding constant . The *P*_cof _value depends only on  and  and not on the binding constant . Therefore, *P*_cof _is indeed a quantitative metric of conformational responsiveness to bound cofactor, uncoupled from the cofactor binding step, and describes the apparent pressure pushing the kinase domain toward the active conformation with the αC-helix "in" position. For ADP, *P*_cof _arises predominantly because of the β-phosphate-magnesium insertion into the kinase domain (β-phosphate latch), which is functionally defined herein using the metal specificity switch approach.

Synthetic molecules lacking a β-phosphate equivalent altogether can achieve the same goal in the absence of Mg^2+ ^ions by inserting bulky moieties in the β-phosphate position. In agreement with this model, sunitinib (Additional file 1, Figure S4), which is predicted to occupy the β-phosphate position only partially [8], has a relatively low *P*_cof _(approximately 30) (Figure 2b).

In CDK2, productive docking of the ATP β-phosphate in the latched position is facilitated by an external effector, cyclin, that stabilizes the αC-helix "in" conformation extrinsically [12]. A conceptually similar mechanism is employed by EGFR, which dimerizes using one EGFR monomer to stabilize another EGFR monomer in the αC-helix "in" conformation [12]. Ire1 does not require a cyclin equivalent, but instead uses homo-oligomerization to stabilize the αC-helix "in" conformation.

## Conclusions

We have characterized an allosteric mechanism by which cofactor binding to the ATP pocket of the Ire1 kinase domain activates the receptor's RNase domain. Our studies of Ire1KR32(E715K) demonstrate that the K702-E715 salt bridge is important for αC-helix positioning and Ire1 activation. In combination, the structural rearrangements in CDK2 (Figure 4) and the presented functional and structural observations with Ire1 converge on a model in which predisposition toward the active conformation of the kinase domain (defined by the position of the αC-helix) is tightly coupled to β-phosphate docking of bound nucleotide (a β-phosphate latch). Suboptimal β-phosphate docking, as in Ire1 ADPβS, Ire1 ATP Mg or CDK2 ATP Mg, does not preclude the nucleotide from binding, but results in the predominantly inactive conformation and, for Ire1, a correspondingly low *P*_cof _value.

For CDK2, Ire1, cSrc, EGFR and perhaps numerous other protein kinases that use αC-helix movement for conformational control, interactions with bound ligands in the ATP pocket may be strongly allosterically coupled to the inactive ↔ active transition and may influence the surface where these kinases bind external effectors. For Ire1, cofactors produce an orders of magnitude shift in the oligomer population, demonstrating that ligands can drive protein-protein interactions involving the kinase/RNase module of Ire1. Analogously, cofactor-mediated conformational tuning of protein kinases related to Ire1 might be deployed to recruit their binding partners and allow protein kinases and catalytically inactive pseudokinases encoded in metazoan genomes to function as signaling conformational switches independent from ATP hydrolysis. The paradoxical in-*trans*-activation of RAF kinase and extracellular signal-regulated kinase by kinase inhibitors [34,35] apparently arises from such a mechanism.

## Methods

### Experimental errors

Observed rate constants were always determined from reaction time courses. Kinetic parameters were reproduced two or more times and had day-to-day variability typically within twofold. This uncertainty is small compared to all effects presented in this work as significant. Errors and experimental uncertainties are indicated where applicable.

### Ire1 expression and purification

Ire1KR32 and its mutants were expressed as glutathione *S*-transferase (GST) fusion proteins using pGEX-6P-2 plasmid (GE Healthcare, Waukesha, WI) and codon-compensated *E. coli *(BL21-CodonPlus(DE3)-RIPL Competent Cells; Stratagene, Santa Clara, CA) as described previously [8]. In brief, expression was performed at room temperature for four hours after isopropyl-β-D-thiogalactopyranoside induction. Cells were lysed using the EmulsiFlex-C3 homogenizer (Avestin Inc. Ottawa, Ontario, Canada) and proteins were purified by performing an affinity column chromatography with subsequent cleavage of the GST tag by using PreScission Protease (GE Healthcare, Waukesha, WI). All protein mutants were fractionated by gel filtration on an S200 column (GE Healthcare, Waukesha, WI) to approximately 99% purity. Proteins stocks (10 to 20 mg/mL) were stored at -80°C in the presence of 5% glycerol.

### RNA substrates

RNA oligonucleotides were purchased from Dharmacon Inc. (Lafayette, CO), labeled at the 5'-terminus using T4 polynucleotide kinase and ^32^P-ATP (PerkinElmer-NEN, Waltham MA) and purified by 20% PAGE that allowed single-nucleotide resolution as described previously [8].

### Ire1 RNase cleavage assay

The kinetics of RNA cleavage were assessed as described previously [8]. Typically, reactions were carried out in a 10-μL volume at 30°C. Reactions were started by adding 1 μL of ^32^P-labeled RNA to 9 μL of premixture containing 20 mmol 4-(2-hydroxyethyl)-1-piperazineethanesulfonic acid (HEPES), pH 7.4, 70 mmol NaCl, 2 mmol MgCl_2 _(unless indicated otherwise), 4 mmol dithiothreitol (DTT), 5% glycerol and cofactors as indicated. The reactions contained ≤ 1 pmol radioactively ^32^P-labeled RNA and were conducted under single-turnover conditions. Unless noted otherwise, the Ire1 concentration was 3 μmol. The enzyme concentration was determined from the Ire1KR32 sequence by using absorbance at 280 nm (e_280 _= 40.8 × 10^3 ^mol^-1^cm^-1^) calculated using the Electronic Lab Notebook program http://biochemlabsolutions.com/ELN/ELN.html. Reactions were quenched at time intervals with 6 μL of stop solution containing 10 mol urea, 0.1% SDS, 0.1 mmol ethylenediaminetetraacetic acid, 0.05% xylene cyanol and 0.05% bromophenol blue. Samples were analyzed by performing 10% to 20% PAGE, and gels were scanned using the Typhoon 9400 scanner (Molecular Dynamics-GE Healthcare, Waukesha, WI) and quantified using ImageQuant image analysis software (Molecular Dynamics-GE Healthcare, Waukesha, WI and GelQuant.NET software http://biochemlabsolutions.com/GelQuantNET.html. The data were plotted and fit in SigmaPlot software (Systat Software Inc., Chicago, IL) to exponential curves to determine observed rate constants and to hyperbolic curves to determine binding constants.

### Optical assay of Ire1 oligomerization

Ire1KR32 oligomerization was assayed in a reaction buffer containing 20 mmol HEPES, pH 7.4, 70 mmol NaCl, 2 mmol MgCl_2_, 4 mmol DTT and 5% glycerol. Cofactors and Mn(CH_3_COO)_2 _were added as indicated for each experiment. The transparency of the samples containing higher concentrations of Ire1 visibly changed immediately upon adding the enzyme. Samples were allowed to sit for 15 minutes to allow for complete Ire1 oligomerization. The OD of the samples was then measured at room temperature (22°C) at 500 nm on a UV-visible spectrophotometer (Amersham Ultrospec 3300 Pro; Amersham-GE Healthcare, Waukesha, WI). OD_500 _was obtained after subtraction of baseline absorbance from the buffer free of Ire1. Sample absorbance did not change upon repeated scans of the same sample, indicating that the oligomerization had reached equilibrium under the conditions of the experiments.

The oligomer of Ire1KR32 has a uniform absorbance over 300 to 800 nm, exhibiting higher absorbance at shorter wavelengths. We selected λ = 500 nm for practical considerations, because at this wavelength proteins do not absorb light and the relevant range of Ire1KR32 concentrations (0.1 to 10 μmol) produces a signal within the optimal instrument range (0.01 to 1 OD). The same titration profiles were obtained not only by reading OD but also by measuring light scattering at a 90° angle at excitation and emission wavelengths of 500 nm on a FluoroLog-3 fluorometer (HORIBA Jobin Yvon, Edison, NJ).

### Ire1KR32 (dCdCdGdCdAdG) complex crystallization

The Ire1KR32 (dCdCdGdCdAdG complex was prepared by mixing Ire1KR32 and dCdCdGdCdAdG (Integrated DNA Technologies, Coralville, IA). When performed at a relatively low NaCl concentration (300 mmol or less), addition of the oligonucleotide caused profound precipitation of the oligomeric complex, as observed upon the addition of ADP Mg [8]. The oligonucleotide thus apparently promoted the formation of Ire1KR32 oligomers analogous to ADP Mg and to APY29. The precipitate readily redissolved upon a slight increase in NaCl concentration, indicating that the oligomerization reaction was salt-dependent and readily reversible. Crystallization was conducted in hanging drops using a stock solution of a premade complex containing Ire1KR32 (12 mg/mL) and 0.6 mmol 2'-deoxy-CCGCAG. The well solution contained 0.12 mol sodium citrate (pH 6.5), 7% PEG 3350 and 4% glucose. Single crystals grew overnight and were cryoprotected in a well solution containing 25% ethylene glycol. The crystals belong to orthorhombic space group C222, distinct from the orthorhombic space group P2_1_2_1_2 previously reported for Ire1KR32 oligomer with bound APY29. Crystallization of a variety of other nucleotides and protein constructs was also tested but produced either inferior crystals or no crystals.

### X-ray data collection and analysis

Diffraction from Ire1 (2'-deoxy-CCGCAG) crystals was collected on Beamline 8.3.1 (Advanced Light Source, Lawrence Berkeley National Laboratory, Berkeley, CA, USA; http://www-als.lbl.gov/index.php/beamlines/beamlines-directory/118-831.html at an X-ray wavelength of 1.115872 Å and an oscillation angle of 1°. The data were indexed, integrated and scaled using the XDS X-Ray Detector software package (MPI for Medical Research, Heidelberg, Germany; http://xds.mpimf-heidelberg.mpg.de/) [36] (Table 1). Five percent of the reflections were marked as a test set (*R*_free_). A molecular replacement solution was found using Phaser Crystallographic Software [37] starting from monomer C of PDB ID 3fbv as a search model. Seven copies of monomer C were found in the asymmetric unit, and the resulting 7-mer of Ire1KR32 was used for rigid body refinement in PHENIX software [38]. Simulated annealing was attempted and, as expected for 6.6-Å resolution, resulted in an excessive separation between residual factor *R*_work _and free residual factor *R*_free _(Table 1). Simulated annealing was therefore used only for calculation of unbiased omit maps (2,000 K). Fourier σ_A_-weighted [39]*F*_obs _- *F*_calc _difference maps were used for interpretation of the parts of the model missing from the starting structure. Electron density and structure were analyzed and graphed in Coot (Crystallographic Object-Oriented Toolkit) software [40] and PyMOL software http://www.pymol.org/. The sevenfold non-crystallographic symmetry (NCS) of the model was used to improve the local quality of the electron density maps by symmetry averaging. However, all elements of Ire1 secondary structure were clearly visible without NCS. Coordinates have been deposited into the Protein Data Bank (PDB:3SDM; http://www.rcsb.org/pdb/search/structidSearch.do?structureId=3SDM.

## Authors' contributions

AVK designed and conducted the biochemical experiments and prepared protein and RNA constructs. AVK and PFE carried out Ire1 crystallization and diffraction data collection. JFM conducted initial diffraction data analyses. AVK and AAK carried out model building and refinement. CZ prepared synthetic Ire1 modulators. PW, KMS and RMS supervised the work. AVK and PW wrote the manuscript. All authors read and approved the final manuscript.

## Supplementary Material

Additional file 1 **Supplementary figures, tables and analyses**. Click here for file

## References

[B1] Ron D Walter P Signal integration in the endoplasmic reticulum unfolded protein response Nat Rev Mol Cell Biol 2007 8 519 529 10.1038/nrm2199 17565364

[B2] Cox JS Walter P A novel mechanism for regulating activity of a transcription factor that controls the unfolded protein response Cell 1996 87 391 404 10.1016/S0092-8674(00)81360-4 8898193

[B3] Shamu CE Walter P Oligomerization and phosphorylation of the Ire1p kinase during intracellular signaling from the endoplasmic reticulum to the nucleus EMBO J 1996 15 3028 3039 8670804 PMC450244

[B4] Sidrauski C Cox JS Walter P tRNA ligase is required for regulated mRNA splicing in the unfolded protein response Cell 1996 87 405 413 10.1016/S0092-8674(00)81361-6 8898194

[B5] Credle JJ Finer-Moore JS Papa FR Stroud RM Walter P On the mechanism of sensing unfolded protein in the endoplasmic reticulum Proc Natl Acad Sci USA 2005 102 18773 18784 10.1073/pnas.0509487102 16365312 PMC1316886

[B6] Pincus D Chevalier MW Aragón T van Anken E Vidal SE El-Samad H Walter P BiP binding to the ER-stress sensor Ire1 tunes the homeostatic behavior of the unfolded protein response PLoS Biol 2010 8 e1000415 10.1371/journal.pbio.1000415 20625545 PMC2897766

[B7] Zhou J Liu CY Back SH Clark RL Peisach D Xu Z Kaufman RJ The crystal structure of human IRE1 luminal domain reveals a conserved dimerization interface required for activation of the unfolded protein response Proc Natl Acad Sci USA 2006 103 14343 14348 10.1073/pnas.0606480103 16973740 PMC1566190

[B8] Korennykh AV Egea PF Korostelev AA Finer-Moore J Zhang C Shokat KM Stroud RM Walter P The unfolded protein response signals through high-order assembly of Ire1 Nature 2009 457 687 693 10.1038/nature07661 19079236 PMC2846394

[B9] Lee KP Dey M Neculai D Cao C Dever TE Sicheri F Structure of the dual enzyme Ire1 reveals the basis for catalysis and regulation in nonconventional RNA splicing Cell 2008 132 89 100 10.1016/j.cell.2007.10.057 18191223 PMC2276645

[B10] Sidrauski C Walter P The transmembrane kinase Ire1p is a site-specific endonuclease that initiates mRNA splicing in the unfolded protein response Cell 1997 90 1031 1039 10.1016/S0092-8674(00)80369-4 9323131

[B11] Getlik M Grütter C Simard JR Klüter S Rabiller M Rode HB Robubi A Rauh D Hybrid compound design to overcome the gatekeeper T338M mutation in cSrc J Med Chem 2009 52 3915 3926 10.1021/jm9002928 19462975

[B12] Zhang X Gureasko J Shen K Cole PA Kuriyan J An allosteric mechanism for activation of the kinase domain of epidermal growth factor receptor Cell 2006 125 1137 1149 10.1016/j.cell.2006.05.013 16777603

[B13] Dar AC Dever TE Sicheri F Higher-order substrate recognition of eIF2α by the RNA-dependent protein kinase PKR Cell 2005 122 887 900 10.1016/j.cell.2005.06.044 16179258

[B14] Dey M Cao C Sicheri F Dever TE Conserved intermolecular salt bridge required for activation of protein kinases PKR, GCN2, and PERK J Biol Chem 2007 282 6653 6660 17202131 10.1074/jbc.M607897200

[B15] Bayliss R Sardon T Vernos I Conti E Structural basis of Aurora-A activation by TPX2 at the mitotic spindle Mol Cell 2003 12 851 862 10.1016/S1097-2765(03)00392-7 14580337

[B16] Yang J Ten Eyck LF Xuong NH Taylor SS Crystal structure of a cAMP-dependent protein kinase mutant at 1.26A: new insights into the catalytic mechanism J Mol Biol 2004 336 473 487 10.1016/j.jmb.2003.11.044 14757059

[B17] Warmus JS Flamme C Zhang LY Barrett S Bridges A Chen H Gowan R Kaufman M Sebolt-Leopold J Leopold W Merriman R Ohren J Pavlovsky A Przybranowski S Tecle H Valik H Whitehead C Zhang E 2-Alkylamino- and alkoxy-substituted 2-amino-1,3,4-oxadiazoles-O-Alkyl benzohydroxamate esters replacements retain the desired inhibition and selectivity against MEK (MAP ERK kinase) Bioorg Med Chem Lett 2008 18 6171 6174 10.1016/j.bmcl.2008.10.015 18951019

[B18] Papa FR Zhang C Shokat K Walter P Bypassing a kinase activity with an ATP-competitive drug Science 2003 302 1533 1537 10.1126/science.1090031 14564015

[B19] Korennykh A Korostelev A Pascal E Finer-Moore J Zhang C Shokat K Stroud R Walter P Structural and functional basis for RNA cleavage by Ire1 BMC Biol in press 10.1186/1741-7007-9-47 PMC3149027 21729333

[B20] Rubio C Pincus D Korennykh A Schuck S El-Samad H Walter P Homeostatic adaptation to endoplasmic reticulum stress depends on Ire1 kinase activity J Cell Biol 2011 193 171 184 10.1083/jcb.201007077 21444684 PMC3082176

[B21] Forconi M Lee J Lee JK Piccirilli JA Herschlag D Functional identification of ligands for a catalytic metal ion in group I introns Biochemistry 2008 47 6883 6894 10.1021/bi800519a 18517225 PMC2758101

[B22] Hougland JL Kravchuk AV Herschlag D Piccirilli JA Functional identification of catalytic metal ion binding sites within RNA PLoS Biol 2005 3 e277 10.1371/journal.pbio.0030277 16092891 PMC1184590

[B23] Hanks SK Hunter T Protein kinases 6. The eukaryotic protein kinase superfamily: kinase (catalytic) domain structure and classification FASEB J 1995 9 576 596 7768349

[B24] Brown NR Noble ME Endicott JA Johnson LN The structural basis for specificity of substrate and recruitment peptides for cyclin-dependent kinases Nat Cell Biol 1999 1 438 443 10.1038/15674 10559988

[B25] Pawson T Kofler M Kinome signaling through regulated protein-protein interactions in normal and cancer cells Curr Opin Cell Biol 2009 21 147 153 10.1016/j.ceb.2009.02.005 19299117

[B26] Ali MM Bagratuni T Davenport EL Nowak PR Silva-Santisteban MC Hardcastle A McAndrews C Rowlands MG Morgan GJ Aherne W Aherne W Collins I Davies FE Pearl LH Structure of the Ire1 autophosphorylation complex and implications for the unfolded protein response EMBO J 2011 30 894 905 10.1038/emboj.2011.18 21317875 PMC3049214

[B27] Jura N Zhang X Endres NF Seeliger MA Schindler T Kuriyan J Catalytic control in the EGF receptor and its connection to general kinase regulatory mechanisms Mol Cell 2011 42 9 22 10.1016/j.molcel.2011.03.004 21474065 PMC3175429

[B28] Brunger AT DeLaBarre B Davies JM Weis WI X-ray structure determination at low resolution Acta Crystallogr D Biol Crystallogr 2009 65 128 133 10.1107/S0907444908043795 19171967 PMC2631637

[B29] Ling H Boodhoo A Hazes B Cummings MD Armstrong GD Brunton JL Read RJ Structure of the shiga-like toxin I B-pentamer complexed with an analogue of its receptor Gb3 Biochemistry 1998 37 1777 1788 10.1021/bi971806n 9485303

[B30] Salt IP Johnson G Ashcroft SJ Hardie DG AMP-activated protein kinase is activated by low glucose in cell lines derived from pancreatic β cells, and may regulate insulin release Biochem J 1998 335 533 539 9794792 10.1042/bj3350533 PMC1219813

[B31] Bradbury DA Simmons TD Slater KJ Crouch SP Measurement of the ADP:ATP ratio in human leukaemic cell lines can be used as an indicator of cell viability, necrosis and apoptosis J Immunol Methods 2000 240 79 92 10.1016/S0022-1759(00)00178-2 10854603

[B32] Martinon F Chen X Lee AH Glimcher LH TLR activation of the transcription factor XBP1 regulates innate immune responses in macrophages Nat Immunol 2010 11 411 418 10.1038/ni.1857 20351694 PMC3113706

[B33] Wiseman RL Zhang Y Lee KP Harding HP Haynes CM Price J Sicheri F Ron D Flavonol activation defines an unanticipated ligand-binding site in the kinase-RNase domain of IRE1 Mol Cell 2010 38 291 304 10.1016/j.molcel.2010.04.001 20417606 PMC2864793

[B34] Poulikakos PI Zhang C Bollag G Shokat KM Rosen N RAF inhibitors transactivate RAF dimers and ERK signalling in cells with wild-type BRAF Nature 2010 464 427 430 10.1038/nature08902 20179705 PMC3178447

[B35] Hatzivassiliou G Song K Yen I Brandhuber BJ Anderson DJ Alvarado R Ludlam MJ Stokoe D Gloor SL Vigers G Morales T Aliagas I Liu B Sideris S Hoeflich KP Jaiswal BS Seshagiri S Koeppen H Belvin M Friedman LS Malek S RAF inhibitors prime wild-type RAF to activate the MAPK pathway and enhance growth Nature 2010 464 431 435 10.1038/nature08833 20130576

[B36] Kabsch W Automatic processing of rotation diffraction data from crystals of initially unknown symmetry and cell constants J Appl Cryst 1993 26 795 800 10.1107/S0021889893005588

[B37] McCoy AJ Grosse-Kunstleve RW Adams PD Winn MD Storoni LC Read RJ *Phaser *crystallographic software J Appl Cryst 2007 40 658 674 10.1107/S0021889807021206 PMC2483472 19461840

[B38] Adams PD Grosse-Kunstleve RW Hung LW Ioerger TR McCoy AJ Moriarty NW Read RJ Sacchettini JC Sauter NK Terwilliger TC PHENIX: building new software for automated crystallographic structure determination Acta Crystallogr D Biol Crystallogr 2002 58 1948 1954 10.1107/S0907444902016657 12393927

[B39] Read RJ Coefficients for maps using phases from partial structures with errors Acta Cryst 1986 A42 140 149

[B40] Emsley P Cowtan K Coot: model-building tools for molecular graphics Acta Crystallogr D Biol Crystallogr 2004 60 2126 2132 10.1107/S0907444904019158 15572765

[B41] Welburn JP Tucker JA Johnson T Lindert L Morgan M Willis A Noble ME Endicott JA How tyrosine 15 phosphorylation inhibits the activity of cyclin-dependent kinase 2-cyclin A J Biol Chem 2007 282 3173 3181 17095507 10.1074/jbc.M609151200

[B42] Weiss MS Global indicators of X-ray data quality J Appl Cryst 2001 34 130 135 10.1107/S0021889800018227

