# Supplementary material for: Cofactor-mediated conformational control in the bifunctional kinase/RNase Ire1
Source: BMC Biol. 2011 Jul 6;9:48. doi: 10.1186/1741-7007-9-48 (PMC3158555; doi:10.1186/1741-7007-9-48)
Supplement: Additional file 1 — Supplementary figures, tables and analyses. [file 1741-7007-9-48-S1.DOC]

# Supplementary materials


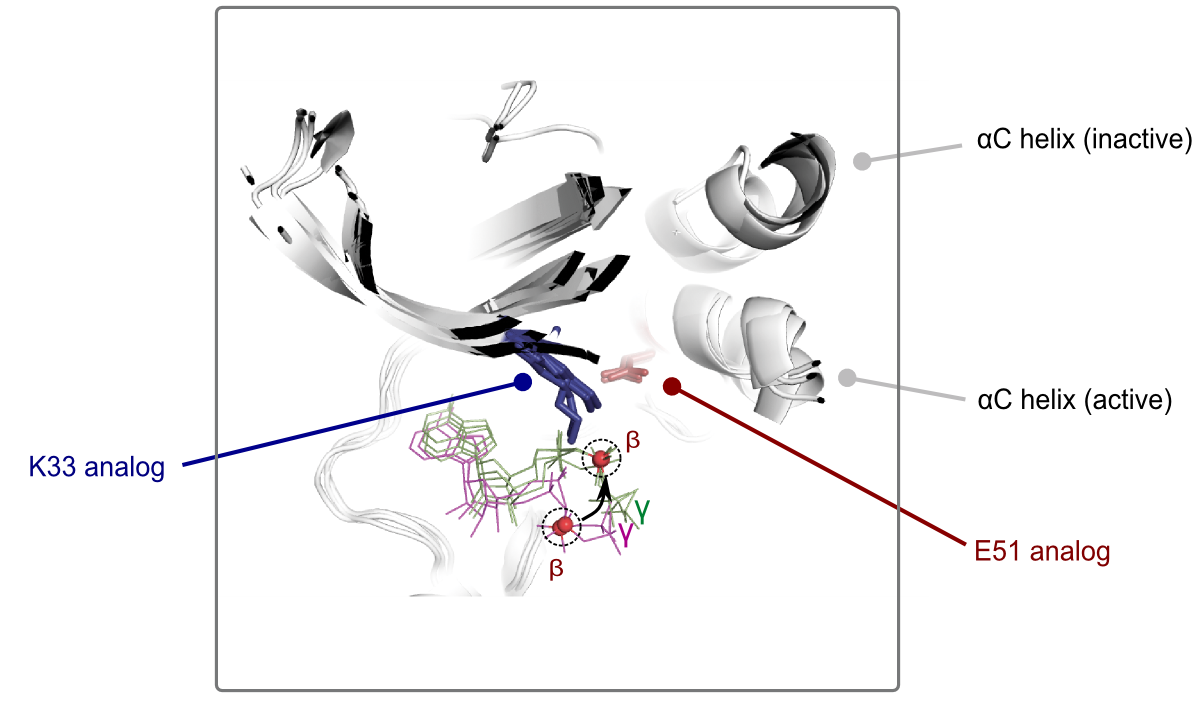

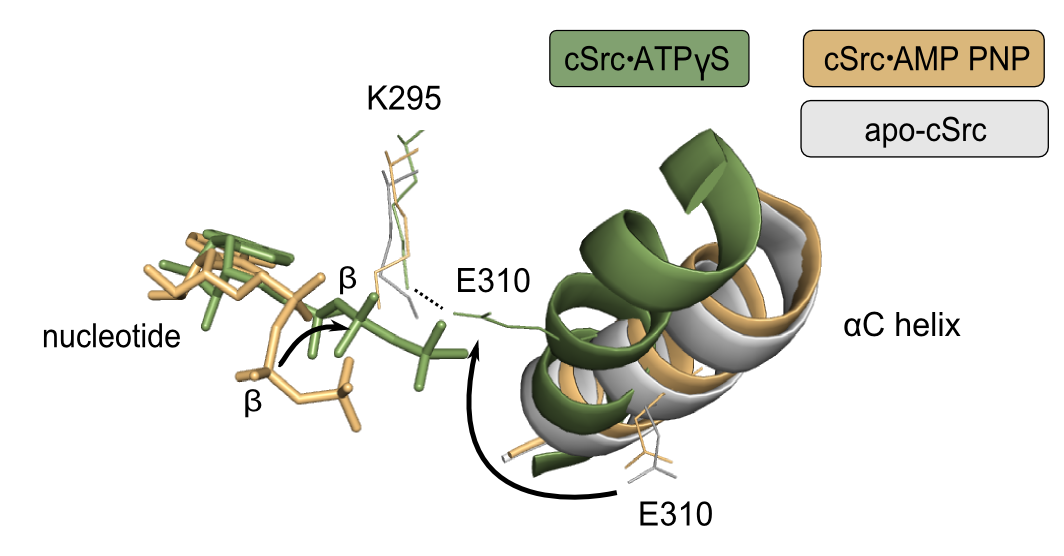


a

b

**Fig. S1.** Comparison of conformations and β-phosphate positions in Ire1-like protein kinases. **a**, Key conformational rearrangements during inactive🡨🡪active transition in cSrc. The movement of the αC-helix and E310 is coupled to the movement of the β-phosphate as in Fig. 4b. The conformational change involves formation of a salt bridge between E310 and K295 and formation of an interaction between K295 and the β-phosphate. Coordinates are from PDB ID: 1fmk (apo-cSrc), 2src (AMP PNP-bound cSrc) and 3dqw (ATPγS-bound cSrc). **b,** Superposition of triphosphate nucleotide-bound Aurora kinase in active conformation (PDB ID 1ol7, PDB ID 2dbw), cAMP kinase in active conformation (PDB ID 1rdq), mitogen-activated kinase MEK1 in inactive conformation (PDB ID 3eqb) and epidermal growth factor receptor EGFR kinase in inactive conformation (PDB ID 3gt8). Two discrete β-phosphate positions in these complexes are indicated. These positions mimic those observed in CDK2 (Fig. 4).


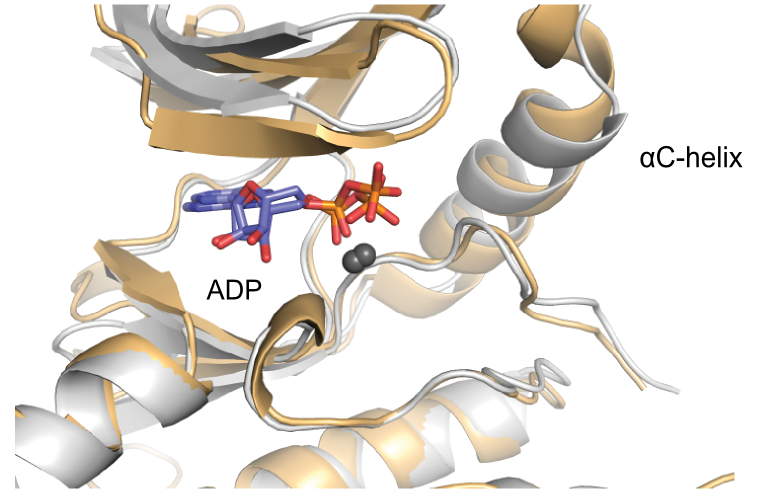

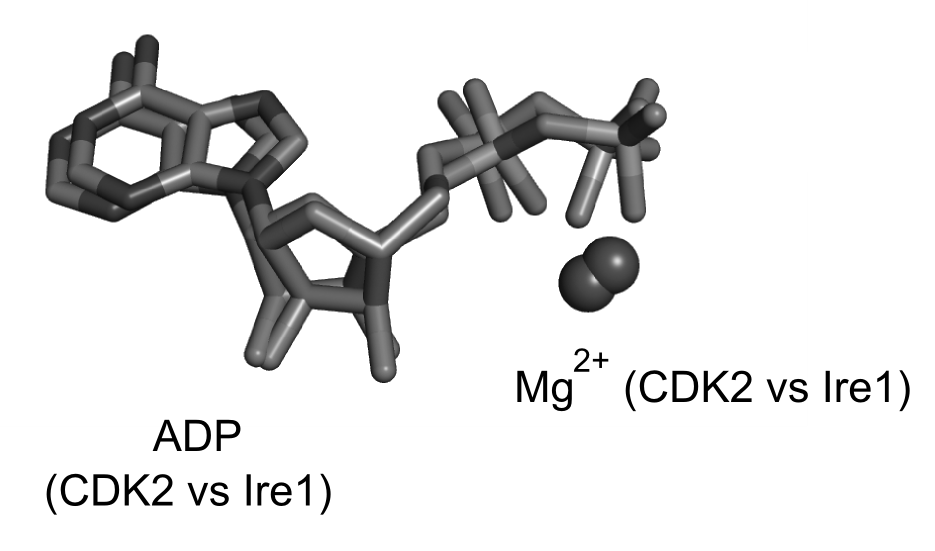


a b

**Fig. S2.** **a**, Superposition of kinase domains of Ire1**•**ADP**•**Mg (tan, PDB ID 2rio) and CDK2**•**ADP**•**Mg (grey, PDB ID 1gy3). **b**, Close-up view of the ADP**•**Mg moiety in the superposition.


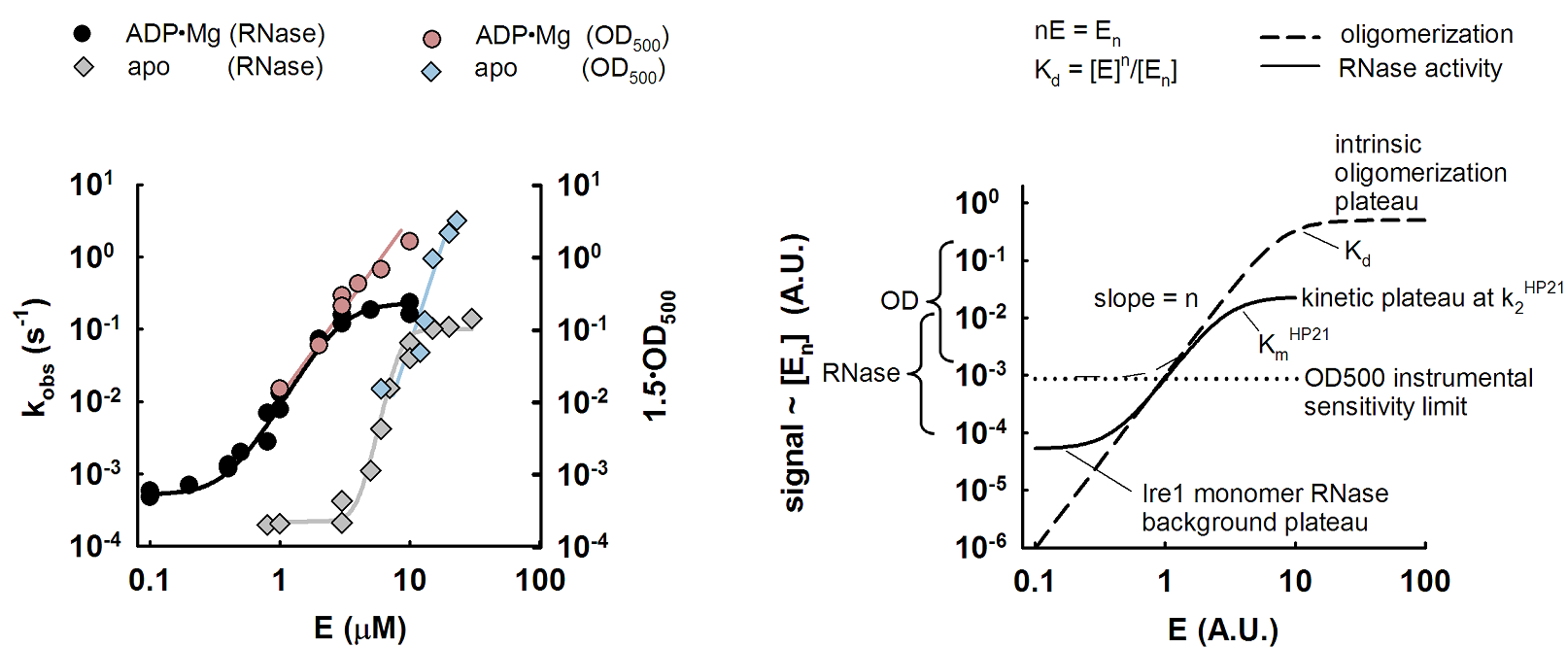


a b

**Fig. S3.** **Linking the RNase and the OD_500_ readouts.** **a**, Superposition of oligomerization profiles determined using RNase activity and OD_500_. To match the profiles, the OD_500_ values were multiplied by a factor of 1.5. This is a valid procedure because OD values do not have physical units and operationally depend on the wavelength used. **b**, Schematic dissection of Ire1 oligomerization profiles. Intrinsic oligomerization equilibrium (solid dashed line), RNase readout (solid line) and OD_500_ readout (solid and narrow dashed lines) are shown. The RNase and the OD_500_ profiles superimpose over a concentration range that exhibits log-linear response in both assays. At high Ire1KR32 concentrations the RNase and the OD_500_ profiles diverge due to the onset of a first-order single-turnover kinetic regime k_2_ for the RNase readout (at E_0_ > K_m_ for HP21). The k_2_ regime is insensitive to enzyme concentration. The OD_500_ trace continues to grow log-linearly above the plateau on the RNase trace, indicating continuing Ire1 oligomerization above 3 μM Ire1KR32. The low-enzyme plateaus in the RNase and the OD_500_ readouts have entirely different origins. In the RNase assay, the plateau occurs due to basal RNase activity of monomeric Ire1KR32, which begins to dominate the overall RNA cleavage rate at low Ire1 dilutions. In the OD_500_ assay, the bottom plateau onsets below ~ 0.01 OD_500_ due to instrument sensitivity limits. The intrinsic oligomerization profile described by the equation K_d_ = [E]n/[E_n_] continues to decrease log-linearly toward infinite dilution (solid dashed line at E→0). The RNase and the OD_500_ readouts complement one another and allow to monitor the uniformly log-linear behavior of the intrinsic oligomerization profile over a broader range of Ire1KR32 concentration than either assay in isolation.


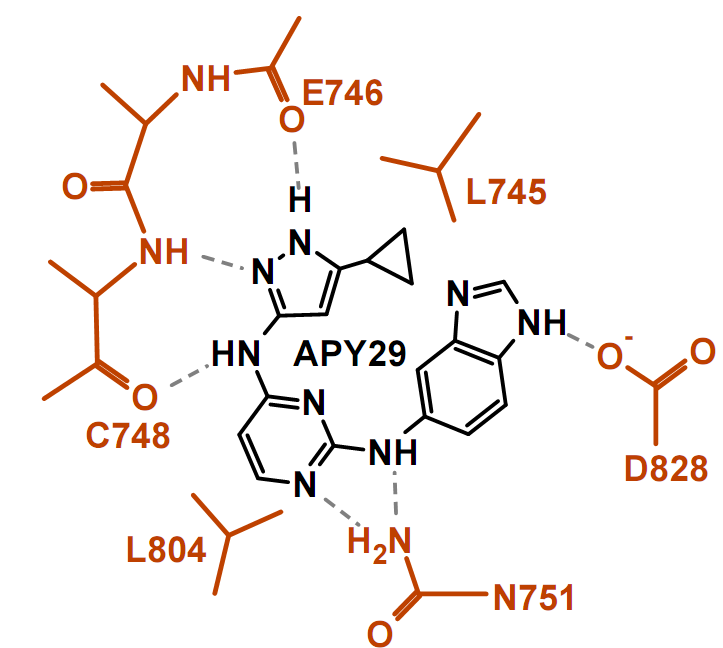

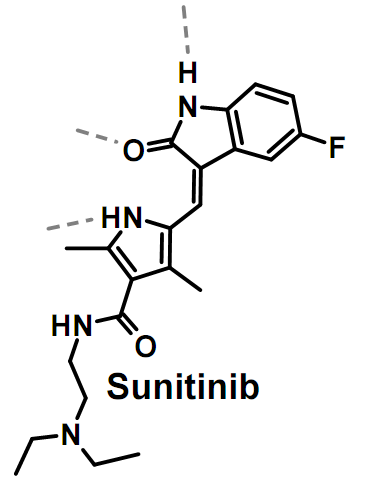

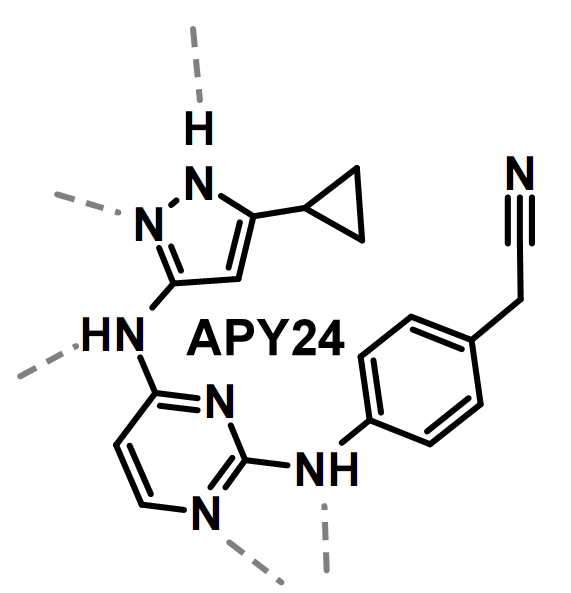


**Fig. S4.** Structures of APY24, APY29 and Sunitinib with interactions in the Ire1 ATP pocket shown by the dashed lines as shown in [1].

**
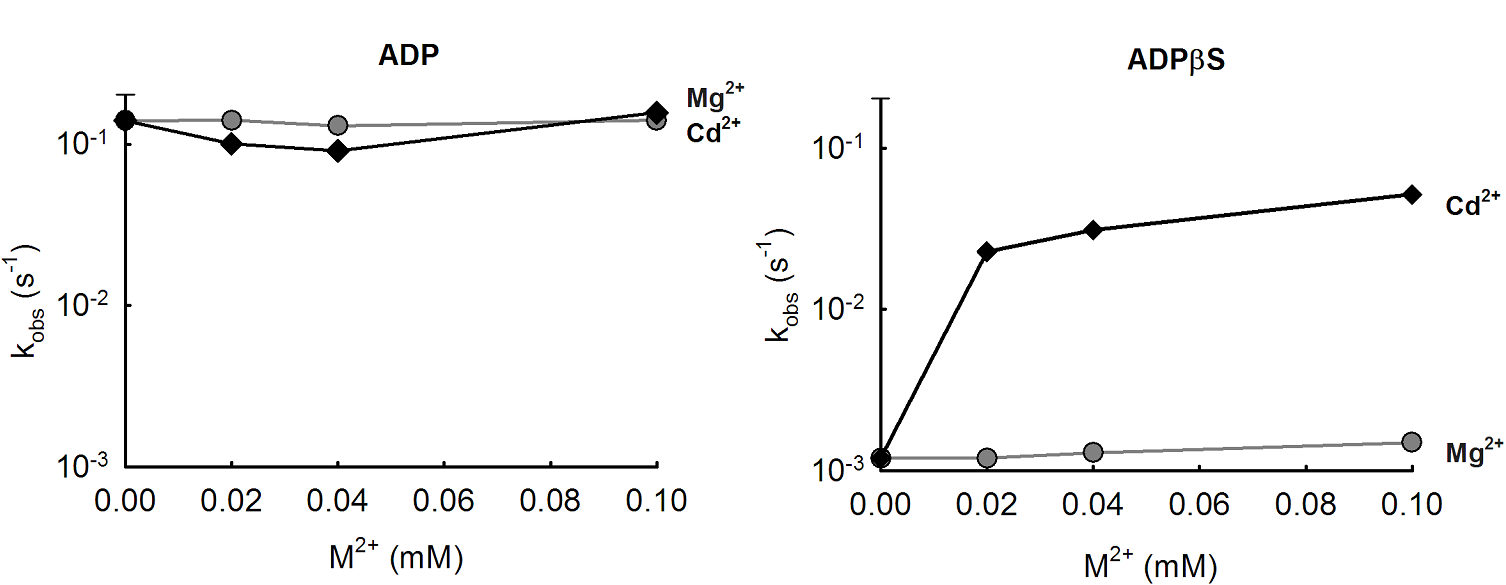
**

**Fig. S5.** **Rescue of the RNA cleavage reaction in the presence of ADPβS by cadmium.** **a**, Effect of Mg^2+^ and Cd^2+^ on the cleavage of HP21 with 3 μM Ire1KR32 in the presence of 2 mM ADP. **b**, Effect of Mg^2+^ or Cd^2+^ on the cleavage of HP21 with 3 μM Ire1KR32 in the presence of 2 mM ADPβS. Reactions contained 0.2 mM Mg^2+^. For undetermined and probably non-specific reasons, cadmium inhibits Ire1 activity at concentrations higher than the range shown here.


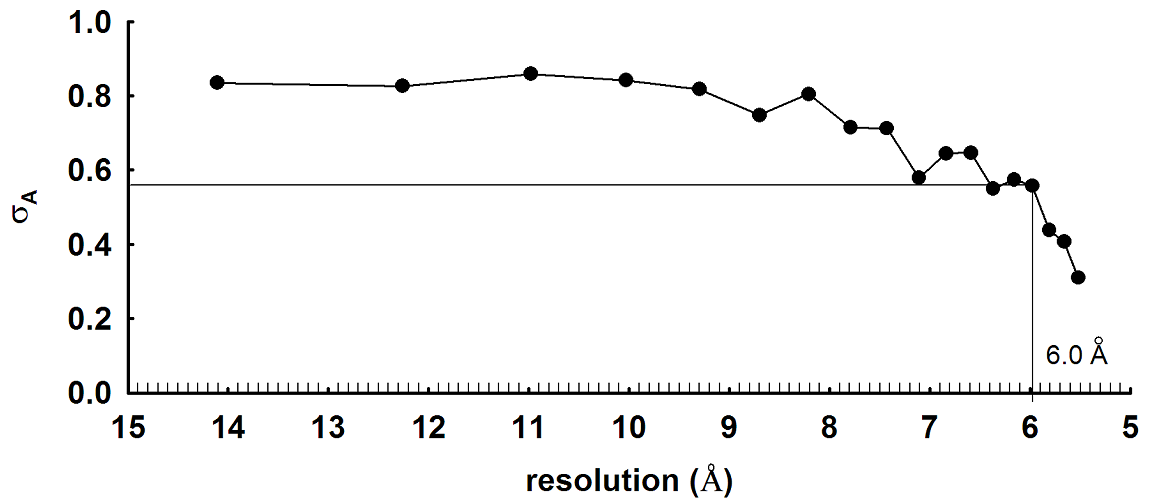


**Fig. S6.** Graph of σ_A_ versus resolution for the C222 crystal [2-3]. Note that values remain significantly above zero at ≥ 6 Å.


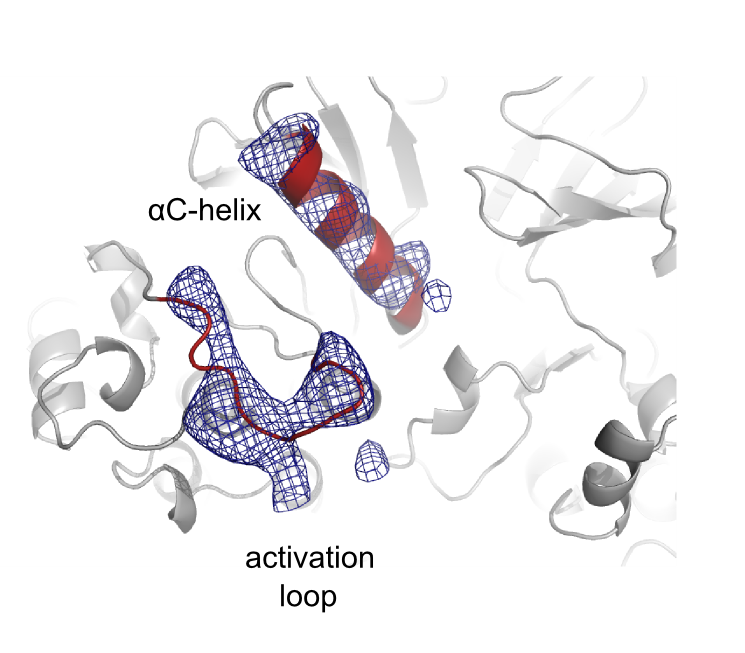


**Fig. S7.** Simulated annealing omit maps F_o_-F_c_ (2σ) for the αC-helix and for the activation loop in the C222 Ire1 oligomer. Both simulated annealing and calculation of maps were conducted without using NCS. Atoms of the αC-helix or the activation loop (red) were deleted from all 7 monomers in the asymmetric unit prior to simulated annealing (2000K) refinement. The secondary structure of the active Ire1 conformation is from the C222 structure after rigid body refinement (Table 1).

# Supplementary Analysis 1


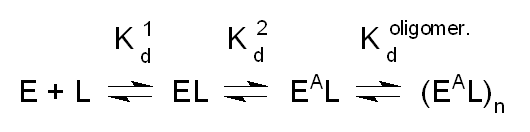


A scheme for Ire1KR32 oligomerization in the presence of cofactors.

**Definitions**

E: Ire1KR32 monomer with αC-helix "out" (inactive) conformation

E_0_: total concentration of Ire1KR32

L: cofactor

L_0_: total concentration of cofactor

EL: Ire1KR32**•**cofactor complex, with inactive conformation of αC-helix

E^A^L: Ire1**•**cofactor complex with active "in" conformation of αC-helix

(E^A^L)_n_: Ire1 oligomer with bound cofactor and active RNase domain

K, K and Kare equilibrium constants defined as in Fig. 6.

**Rate equation**

In the presence of ADP or other potent cofactors, the RNase activity of Ire1 arises from the oligomer (E^A^L)_n_. The log-linear cooperative activation profile (Fig. 1a) is obtained under single-turnover conditions and, for the most part, with sub-saturating RNA and Ire1 concentrations, where the rate of RNA cleavage is proportional to the equilibrium concentration of oligomer (E^A^L)_n_:

V ~ [(E^A^L)_n_] (1)

From the definition of K= $\frac{\text{[E}^{\text{A}}\text{L}]^{\text{n}}}{\text{[(E}^{\text{A}}\text{L)}_{\text{n}}\text{]}}$it follows that:

V ~ [E^A^L]n/ K (2)

The RNase activity will follow the dependence of [E^A^L]^n^ on co-factor concentration. When concentration of ligand is in excess over Ire1 and Ire1 oligomer is a minor Ire1 species (L_0_ >> E_0_ and E_0_ << K, which is true below ~ 10 μM Ire1, as suggested by the log-linear dependence of optical density on E_0_ at such concentrations, Fig. 1b), it is possible to obtain in analytical form the dependence of [E^A^L] on the microscopic rate constants. Using equations (3) and (4),

E_0_ = [E] + [EL] + [E^A^L] (3)

L_0_ ≈ L (4)

equation (5) is obtained:

[E^A^L] = (5)

Limits of equation (5) can be used to evaluate the experimentally measured parameters K_cof_ and P_cof_ via the microscopic constants K, K.

**Expression of P_cof_ via microscopic equilibrium parameters**

The parameter P_cof_ is measured at saturating ligand concentrations (L_0_ >> K). From equation (5) it follows that reaction rate saturates (as observed in Fig. 2a) when L_0_ >> K**•**K. Therefore, at the plateau in Fig. 2a:

[E^A^L] ≈ $\bullet\frac{1}{1+\text{K}_{\text{d}}^{2}}$ (6)

Combining equations (2) and (6) results in the relationship between the rate constant measured at saturating ligand concentrations (which is linearly proportional to P_cof_) and the microscopic equilibrium constant K (7):

P_cof_ ~ V~ $\frac{\text{E}_{0}^{\text{n}}}{\text{K}_{\text{d}}^{\text{oligomer.}}\bullet(1+\text{K}_{\text{d}}^{2})^{\mathrm{n}}}$ ~ $\frac{1}{\text{K}_{\text{d}}^{\text{oligomer.}}\bullet(1+\text{K}_{\text{d}}^{2})^{\text{n}}}$ (7)

From equation (7) it follows that P_cof_ depends not only on K, but also on K, E_0_ and on the cooperativity coefficient n. The dependence of P_cof_ on K and K but not on K suggests that P_cof_ is linked to the conformational properties of cofactor-bound Ire1 monomer but not to the cofactor binding constant. Both K and K characterize the conformational properties of cofactor-bound Ire1. K reflects the ability of cofactors to move the αC-helix into the active position, whereas K reflects the ability of oligomer to form from monomers with bound cofactor and should also depend on the conformation of such monomers. Notably, P_cof_ cannot increase indefinitely as better and better cofactors are added. An upper limit of P_cof_ is observed when bound cofactor leads to a K << 1. Different reference conditions (deviating from E_0_ = 3 µM) can be defined, if needed.

**Expression of K_cof_ via microscopic equilibrium parameters**

Experimentally measured binding constant K_cof_ is classically defined as concentration of cofactor at which ½ maximum rate of RNA cleavage is observed. From equations (2) and (5), the concentration of L_0_ providing ½ maximum rate can be determined analytically (8):

L_0_ ^½ maximum^ = K_cof_ = $\frac{\text{K}_{\text{d}}^{1}\bullet\text{K}_{\text{d}}^{2}}{1+\text{K}_{\text{d}}^{2}}$ (8)

From equation (8) it follows that K_cof_ is a composite constant proportional to the microscopic binding constant K and the conformational constant K. For cofactors that activate Ire1 weakly (K >> 1), K_cof_ probably represents the true microscopic affinity of a ligand for Ire1. For cofactors that bias Ire1 strongly toward the active conformation (K << 1), K_cof_ appears smaller than the microscopic constant K.

**References**

1. Korennykh AV, Egea PF, Korostelev AA, Finer-Moore J, Zhang C, Shokat KM, Stroud RM, Walter P: **The unfolded protein response signals through high-order assembly of Ire1**. *Nature* 2009, **457**(7230):687-693.

2. Brunger AT, DeLaBarre B, Davies JM, Weis WI: **X-ray structure determination at low resolution**. *Acta Crystallogr D Biol Crystallogr* 2009, **65**(Pt 2):128-133.

3. Ling H, Boodhoo A, Hazes B, Cummings MD, Armstrong GD, Brunton JL, Read RJ: **Structure of the shiga-like toxin I B-pentamer complexed with an analogue of its receptor Gb3**. *Biochemistry* 1998, **37**(7):1777-1788.
